# HLA-B27, Mantoux Test, and Eosinophil Count: Help in the Diagnosis of Reactive Arthritis Due to Tubercular Hypersensitivity

**DOI:** 10.7759/cureus.95386

**Published:** 2025-10-25

**Authors:** Akshita Makkar, Venkatesh S Pai, Prasan K Panda

**Affiliations:** 1 Internal Medicine, All India Institute of Medical Sciences, Rishikesh, Rishikesh, IND

**Keywords:** eosinophilia, exudative pleural effusion, mantoux positivity, nonsteroidal anti-inflammatory drugs, tuberculosis preventive therapy

## Abstract

Reactive arthritis (ReA) is a form of inflammatory arthritis usually triggered by gastrointestinal/genitourinary infections. We present the case of a middle-aged man with HLA-B27-associated ReA and tuberculosis (TB) hypersensitivity, manifesting as polyarthritis, inflammatory low back pain, and pleural effusion. The patient had a three-year history of progressive joint pain, initially involving the knees, followed by the bilateral ankle joints and small joints of the hands, wrists, shoulders, elbows, and axial skeleton. He also had a prolonged fever with an exudative pleural effusion, eosinophilia, and Mantoux positivity, though pleural fluid adenosine deaminase (ADA) and cartridge-based nucleic acid amplification test (CBNAAT) were within normal limits and negative, respectively. Due to the autoimmune-inflammatory overlap, treatment with nonsteroidal anti-inflammatory drugs (NSAIDs) resulted in rapid improvement in joint pains, and tuberculosis preventive therapy was initiated to prevent TB reactivation. The case highlights the complex interaction between immune-mediated inflammation and TB hypersensitivity, emphasizing the need for careful differential diagnosis and treatment plans in seronegative arthritis with systemic features.

## Introduction

The term "reactive arthritis" (ReA) was first coined in 1969 to denote a non-septic inflammatory arthritis occurring as a sequela of a remote infection, most commonly involving the gastrointestinal or urogenital tract [1]. This condition is characterized by symptoms such as asymmetric oligoarthritis, enthesitis, and extra-articular manifestations, including conjunctivitis and urethritis [2]. Over time, the detection of microbial antigens, such as *Salmonella* and *Yersinia lipopolysaccharides*, as well as microbial deoxyribonucleic acid (DNA) and ribonucleic acid (RNA), and, in some cases, viable *Chlamydia* species within joint tissue, has complicated the distinction between ReA and post-infectious arthritis. Despite these findings, there are currently no universally accepted or validated diagnostic criteria. Consequently, diagnosis is predominantly clinical and is based on the presence of acute oligoarticular arthritis, typically affecting large joints, which develops within 2 to 4 weeks of a preceding infection [3]. The pathogenesis of ReA involves immune dysregulation following a gastrointestinal/genitourinary infection, with the Human Leukocyte Antigen-B27 (HLA-B27) allele playing a pivotal role in enhancing susceptibility to post-infectious arthritis.

The Mantoux test (also called the tuberculin skin test) is a diagnostic tool used to detect whether a person has been exposed to *Mycobacterium tuberculosis*, the bacterium that causes tuberculosis (TB). However, its limitations are false positives if Bacillus Calmette-Guérin (BCG) vaccinated and *Nontuberculous Mycobacteria* (NTM) infected and false negatives if immunosuppressed and having miliary TB. A positive Mantoux test also does not distinguish between latent and active or remote and recent TB; it indicates only a prior exposure to the *Mycobacterium tuberculosis* (MTB) pathogen [4]. In some cases, TB can trigger immune-mediated reactions such as ReA, complicating the diagnostic process when both conditions coexist [5-7]. The immune response to latent TB in susceptible individuals, particularly those with a history of TB exposure, can manifest as hypersensitivity reactions, including inflammation of joints, skin, and other tissues [8].

Eosinophilia, characterized by an elevated eosinophil count, is often associated with hypersensitivity reactions, including those linked to TB. Although eosinophilia is more commonly seen in parasitic infections and allergic conditions, it can also be a feature of immune responses in ReA and/or TB-related hypersensitivity disorders [9,10]. The complexity of diagnosing TB-related autoimmune and inflammatory conditions increases in patients who present with overlapping symptoms of TB exposure and autoimmune arthritis. However, a few terminologies need clarification before further case discussion: 'Autoimmune-inflammatory overlap cases,' where features of autoimmunity coexist with infection-related inflammation; 'TB hypersensitivity,' immune-mediated manifestations triggered by latent or prior TB exposure; 'Tubercular ReA,' a ReA phenotype triggered by mycobacterial antigens; Lastly, another related confusing term is 'Poncet’s disease,' a sterile, non-erosive arthritis associated with active TB, without direct joint invasion. Understanding the intricate relationship between these terms is essential for the accurate diagnosis and management of patients with such complex presentations.

This case report explores these interplays of HLA-B27, the Mantoux test, and eosinophil count, and how they may help in the diagnosis of ReA due to TB hypersensitivity.

## Case presentation

A 45-year-old man presented with a three-year history of progressive pain and swelling in both knee joints involved sequentially, later involving the bilateral wrists, metacarpophalangeal (MCP), and proximal interphalangeal (PIP) joints, with sparing of the distal interphalangeal (DIP) joints. The symptoms subsequently extended to the shoulders, elbows, and both sacroiliac joints. He reported early morning stiffness (EMS) of both the lower back and small joints of the hands lasting 1.5 hours, improving with activity but worsening with rest, with significant nocturnal exacerbation of symptoms.

Over the last five months, he experienced intermittent fever of up to 103°F, occurring 2-3 times per day, associated with chills and rigor, but without evening rise or night sweats. He also developed a cough with whitish expectoration for 15 days (15-20 mL/day), without hemoptysis, along with bilateral anterior and posterior pleuritic chest pain, described as a sharp stabbing pain while coughing on a background of constant dull aching continuous pain over the right infra-axillary area. There was no history of abdominal pain, burning micturition, diarrhea, or cutaneous pus discharge. He also denied any features suggestive of other connective tissue diseases.

On examination, the patient was afebrile and tachycardic at 114 beats/min, with a BP of 118/75 mm Hg and a SpO2 of 99% on room air. A musculoskeletal examination was performed, eliciting a tender joint count (TJC) of 28 and swollen joint count (SJC) of 6, with a positive asymmetric Flexion ABduction External Rotation test (FABER) test (L > R), suggesting sacroiliitis. Respiratory examination revealed right-sided reduced vocal fremitus, decreased air entry, diminished vocal resonance, and decreased breath sounds in the right infra-axillary region, suggesting pleural involvement. Other systems were within normal limits.

Given the asymmetrical, additive, inflammatory peripheral arthritis with axial disease in the form of sacroiliitis, the most likely diagnosis was spondyloarthritis (SpA), peripheral + axial, including ankylosing spondylitis, reactive arthritis, psoriatic arthritis, or enteropathic arthritis. Of these differentials, the absence of both a personal/family history of psoriasis and inflammatory bowel disease-related signs/symptoms, along with having majorly symmetrical joint involvement, made these diagnoses very unlikely. The fever with pleural effusion and asymmetric sacroilitis raised suspicion for an infectious or inflammatory etiology. Other differentials included tubercular effusion with Poncet's, rheumatoid arthritis with empyema, adult-onset Still’s disease (fever with arthritis), and connective tissue disorders. The pleural effusion necessitated ruling out TB pleuritis, malignancy, and inflammatory effusions.

**Figure 1:**
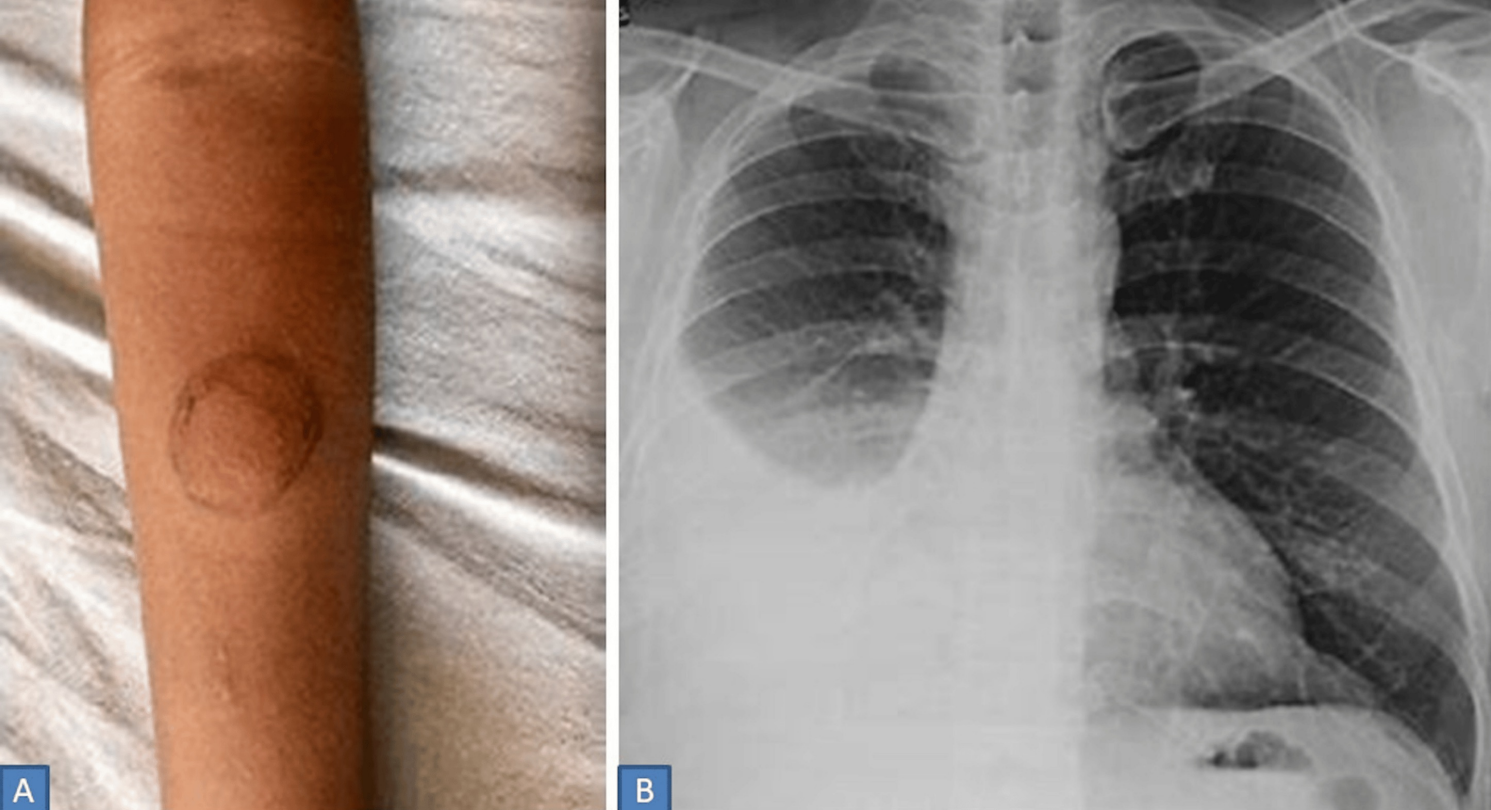
Clinico-radiological images. Patient showing positive Mantoux test with induration of 22 mm² (A). Chest X-ray Postero-anterior view showing right-sided pleural effusion with otherwise normal parenchyma (B).

Initial blood tests revealed normocytic normochromic anemia, eosinophilia, and elevated inflammatory markers (ESR: erythrocyte sedimentation rate, CRP: C-reactive protein), with normal liver and kidney function tests (Table 1). The Mantoux test was positive (Figure 1A), and chest X-ray and computed tomography (CT) suggested right-sided pleural effusion (Figure 1B). Pleural fluid analysis revealed exudate by Light's criteria with normal adenosine deaminase (ADA) and negative Gram stain, potassium hydroxide (KOH), cartridge-based nucleic acid amplification test (CBNAAT), and cultures (Table 2). Also, the rheumatoid factor was borderline positive (16 IU/ml), while anti-cyclic citrullinated peptide (anti-CCP) was negative. Magnetic resonance imaging (MRI) of the sacroiliac and hip joints came out normal. Bronchoscopy and bronchoalveolar lavage (BAL) analysis, including CBNAAT, came out to be negative for any pathogenic organism.

**Table 1:** Basic investigations of the patient during hospitalization. Hb: Hemoglobin, TLC: Total Leukocyte Count, DLC: Differential Leukocyte Count, ALT: Alanine Transaminases, AST: Aspartate Transaminases, ALP: Alkaline Phosphatase, GGT: Gamma Glutamyl Transferase, ESR: Erythrocyte Sedimentation Rate, CRP: C-Reactive Protein, LDH: Lactate Dehydrogenase.

Investigation	SI Unit	Reference Range	During admission
Hb	g/dL	13-17	10.6
TLC	×10³ cells/mm³	4-11	7.29
DLC (Neutrophils/Lymphocytes /Monocytes/Eosinophils)	%	40-70/20-40/2-8/0-2	54.4/27.8/6.6/11.2
Platelet count	×10³ cells/mm³	150-400	329
Total Bilirubin	mg/dL	0.3 – 1.2	0.32
Direct Bilirubin	mg/dL	0 – 0.2	0.10
ALT	U/L	0-50	24
AST	U/L	0-50	23
ALP	U/L	30-120	105
GGT	U/L	0-55	30
Total Protein	g/dL	6.6-8.3	6.9
Albumin	g/dL	3.5-5.2	3.7
Globulin	g/dL	2.5-3.2	3.2
Urea	mg/dL	17-43	30
Creatinine	mg/dL	0.72-1.18	0.73
ESR	mm/hr	0-15	65
CRP	mg/dL	0-1	120
LDH	U/L	140-280	326

**Table 2:** Advanced investigations of the patient during hospitalization. RF: Rheumatoid Factor, Anti-CCP: Anti-Cyclic Citrullinated Peptide, HLA-B27 (PCR): Human Leukocyte Antigen B27 (Polymerase Chain Reaction), CECT: Contrast-Enhanced Computed Tomography, MRI: Magnetic Resonance Imaging, BAL: Bronchoalveolar Lavage, TLC: Total Leukocyte Count, DLC: Differential Leukocyte Count, ADA: Adenosine Deaminase, LDH: Lactate Dehydrogenase.

Investigations during admission		SI Unit	Reference Range	Result
RF		IU/ml	< 15	16 (Positive)
Anti-CCP		U/mL	< 20	Negative
HLA B27 (PCR)		-	Normally absent/negative	Positive
CECT (Thorax +Abdomen)		-	-	Right sided loculated pleural effusion
MRI sacro-iliac (SI) & bilateral hip joints		-	-	B/L SI joints appear normal with maintained joint space. Well-defined lesion with internal hypointense nidus in head of right femur - likely osteoid osteoma
Bronchoscopy + BAL		-	-	No nodularity or granuloma found. Few pus cells, no microorganisms including KOH, CBNAAT- Negative
Pleural fluid analysis	TLC	cells/µl	< 1000 (usually < 300)	1650
	DLC (Neutrophils/ Lymphocytes)	%	Predominantly mononuclear cells (lymphocytes, mesothelial cells); Neutrophils < 25%	2-98
	Glucose	mg/dl	Similar to blood glucose; typically 60–100	57 (Corresponding blood glucose - 120)
	Protein	mg/dl	< 3.0	5.7
	ADA	U/L	< 40	24.98
	LDH	U/L	< 200	206

The patient had a chronic, asymmetrical, inflammatory, non-erosive, non-deforming polyarthritis with non-radiographic sacroiliitis, for which he was started on non-steroidal anti-inflammatory drugs (NSAIDs) - Naproxen (250 mg twice daily), leading to significant improvement in joint pain.

Given his positive Mantoux test, HLA-B27 positivity, and respiratory signs and symptoms, he was initiated on tuberculosis preventive therapy (TPT, Isoniazide 5 mg/kg/day for nine months).

After a week of NSAID therapy, the patient demonstrated substantial relief in joint pain. His TJC progressively decreased, and ESR and CRP showed a declining trend at discharge (TJC-8, SJC-4, ESR-23 mm/hr, CRP-43 mg/dL). At subsequent follow-up at one month, a minimal residual, non-tappable pleural effusion was noted, likely resolving.

## Discussion

The interplay between HLA-B27-associated reactive arthritis and TB hypersensitivity represents a unique diagnostic challenge. HLA-B27 is a genetic marker strongly associated with increased susceptibility to autoimmune conditions, particularly spondyloarthritis, which includes reactive arthritis [1,2]. ReA commonly occurs following infections such as Chlamydia, Salmonella, and Yersinia, but it has also been described in patients with respiratory/mycobacterial infection, where it manifests as a hypersensitivity reaction [6,11-14]. The presence of symmetrical polyarthritis, eosinophilia, and a positive Mantoux test can suggest an overlap of these conditions, leading to diagnostic confusion.

It is considered that there are two forms of ReA. A classification into HLA-B27-associated and non-associated forms has also been proposed [3]. Post-streptococcal, Lyme, and viral arthritis are HLA-B27 non-associated and should be described as distinct entities under the general heading of ‘infection-related arthritides.’ Three central aspects of the pathogenesis of ReA are the presence of bacteria or bacterial products in the joint, the bacterium-host interaction, and the local immune response directed against these bacteria. The arthritogenic peptide hypothesis suggests that the arthritis is triggered by a T-cell response to specific antigenic peptides derived from the triggering bacteria and that these T cells then cross-react with self-antigen-derived peptides. Certain bacteria have constituents that display strong homology to proteins of the host (e.g., YopH of Y. pseudotuberculosis and CD45). This molecular mimicry can give rise to tolerance of some microbes, which may thus escape from the immune system of the host. On the other hand, mimicry may also be differently interpreted by the host, because the homology between this bacterial constituent and an antigen of the articular cavity can induce an ‘autoimmune’ synovitis [15].

Among the differentials, SPA (ReA) due to TB hypersensitivity is the most likely diagnosis. The closest differential of TB effusion with Poncet’s was kept behind. Poncet disease requires an active tubercular focus, which we were not able to prove despite invasive testing like pleural fluid and BAL analyses, as neither the ADA of pleural fluid was positive, nor could we microbiologically prove TB in both fluids. With the presence of sacroiliac and axial involvement, the diagnosis of Poncet’s arthritis was ruled out as per the Sharma and Pinto diagnostic criteria [16]. Also, classically, Poncet's disease was reported as arthritis developing in the acute onset of tuberculosis and ameliorating with anti-tuberculosis treatment without causing joint destruction. Non-erosive asymmetrical arthritis, borderline positive RF (can be a false positive), and negative anti-CCP made rheumatoid arthritis less likely. Adult-onset Still's disease (AOSD) was also less likely, as only two major criteria (fever and arthritis) were present, with no minor, thus not fulfilling Yamaguchi's criteria, including the fact that there was another diagnosis that could explain the symptoms. 

TB hypersensitivity can present with delayed-type hypersensitivity reactions, in which activated T cells and other immune mediators induce inflammation in response to mycobacterial antigens [17,18]. These immune responses can lead to the development of musculoskeletal symptoms, including arthritis, which may mimic ReA. In fact, several studies have reported that TB-induced arthritis can present with features similar to ReA, particularly in patients with a history of latent TB exposure [8]. The diagnosis of TB-related arthritis often relies on the exclusion of active TB, which can be challenging in the absence of clear radiological or microbiological evidence.

In this context, eosinophilia plays a significant role in identifying immune-mediated processes. Elevated eosinophil counts can be a marker of TB hypersensitivity, as eosinophils are involved in the immune response to mycobacterial antigens. In TB-related hypersensitivity, eosinophils contribute to tissue inflammation and damage, leading to symptoms such as arthritis [9]. However, eosinophilia is rarely observed in other autoimmune diseases; hence, its presence supports TB hypersensitivity, as in this case [10].

Tuberculous pleural effusion (TPE) is a common finding in extrapulmonary tuberculosis, resulting from a combination of direct infection of the pleural space by *Mycobacterium tuberculosis* and a delayed hypersensitivity reaction to mycobacterial antigens. This immunological response leads to an exudative pleural effusion characterized by elevated protein levels and a predominance of lymphocytes in the pleural fluid. Clinically, TPE often presents acutely with symptoms such as fever, cough, and pleuritic chest pain. The pleural fluid is typically an exudate with a high lymphocyte count. The pathogenesis of TPE involves both the direct presence of the bacteria in the pleural space and the body's immune response to these antigens, leading to the characteristic exudative effusion [19-21].

Hence, ReA can be presented by tuberculosis as a triggered hypersensitivity reaction. The symptoms of ReA usually begin 2-4 weeks after the previous infection. The average duration of arthritis is 4-5 months, but two-thirds of patients may experience musculoskeletal symptoms that can persist for more than one year [3,12]. Reactive arthritis classically presents with an oligoarthritis, favoring the large joints of the lower extremities. However, involvement of the joints ranges from a transient monoarthritis to a widespread polyarthritis involving the peripheral and axial joints with or without characteristic extra‐articular lesions, particularly enthesopathy, psoriasiform mucosal and cutaneous lesions, inflammatory eye disease, and cardiovascular lesions (aortitis-aortic root dilatation, valvular lesions-aortic regurgitation, conduction abnormalities-atrio-ventricular blocks, and rarely pericarditis). In this case study, a few limitations were present: the incubation period from the preceding tubercular infection was not known, the duration of illness was much prolonged, the fever was persistent, and lastly, the polyarticular type of involvement.

The management of HLA-B27-associated ReA with TB hypersensitivity involves a careful balance of immunosuppressive therapies and TB preventive treatment. NSAIDs, such as naproxen, 250 mg twice daily after food for 2-4 weeks, are frequently used to manage the inflammation and pain associated with arthritis [22]. However, in patients with a history of latent TB, anti-tubercular therapy (ATT) may be necessary if active TB is suspected. Tuberculosis preventive treatment (TPT) is recommended for individuals with latent TB, and it helps reduce the risk of reactivation [23]. The decision between TPT and ATT is often based on the clinical, radiological, and microbiological evaluation of TB activity, determined by evidence of TB activity, not arthritis severity [24].

## Conclusions

Clinicians must remain vigilant to the possibility of reactive arthritis due to TB hypersensitivity, particularly in endemic regions. Mantoux test positivity and hypereosinophilia suggest TB hypersensitivity. ReA due to TB hypersensitivity can present with fever, chronic polyarthritis, eosinophilia, and exudative pleural effusion. In the absence of active TB (microbiologically proven), TB preventive therapy is typically recommended in this setting to reduce the risk of reactivation.
